# Severe Infection in Anti-Glomerular Basement Membrane Disease: A Retrospective Multicenter French Study

**DOI:** 10.3390/jcm9030698

**Published:** 2020-03-04

**Authors:** Pauline Caillard, Cécile Vigneau, Jean-Michel Halimi, Marc Hazzan, Eric Thervet, Morgane Heitz, Laurent Juillard, Vincent Audard, Marion Rabant, Alexandre Hertig, Jean-François Subra, Vincent Vuiblet, Dominique Guerrot, Mathilde Tamain, Marie Essig, Thierry Lobbedez, Thomas Quemeneur, Jean-Michel Rebibou, Alexandre Ganea, Marie-Noëlle Peraldi, François Vrtovsnik, Maïté Daroux, Adnane Lamrani, Raïfah Makdassi, Gabriel Choukroun, Dimitri Titeca-Beauport

**Affiliations:** 1 Department of Nephrology, Dialysis, and Transplantation, University of Picardie Jules Verne, Amiens University Hospital, F-80054 Amiens, France; 2 Department of Nephrology, CHU Pontchaillou, University Hospital, Rennes, France and University of Rennes 1, Institut National de la Santé Et de la Recherche Médicale (INSERM) U1085-IRSET, F-35000 Rennes, France; 3 Department of Nephrology and Immunology, University François-Rabelais, Bretonneau Hospital, CHU Tours and EA4245, F-37044 Tours, France; 4 Nephrology Department, CHU of Lille, University of Lille, UMR 995, F-59000 Lille, France; 5 Department of Nephrology, Georges Pompidou European Hospital, Assistance Publique-Hôpitaux de Paris (APHP), F-75015 Paris and INSERM UMRS970, F-92100 Boulogne-Billancourt, France; 6 Department of Nephrology, Dialysis and Renal Transplantation, University Hospital of Grenoble, F-38700 Grenoble, France; 7 Department of Nephrology, Edouard Herriot Hospital, Hospices Civils de Lyon, Carmen INSERM 1060 and Univ Lyon, F-69437 Lyon, France; 8 Department of Nephrology and Renal Transplantation, Reference Center-Idiopathic Nephrotic Syndrome, Henri-Mondor Hospital/Albert-Chenevier, APHP F-94000 Créteil, INSERM U955, Paris Est Créteil University, F-94000 Créteil, France; 9 Pathology Department, Necker University Hospital, AP-HP. Centre-Université de Paris, F-75015 Paris, France; marion.rabant@aphp.fr; 10 Sorbonne University, APHP, Renal Intensive Care Unit, Tenon Hospital, F-75020 Paris, France; 11 Department of Nephrology, Dialysis and Transplantation, University Hospital, Angers and CRCINA, INSERM, Université de Nantes, Université d’Angers, F-49100 Angers, France; 12 Department of Nephrology and Renal Transplantation, Reims University Hospital, F-51092 Reims, France; 13 Department of Nephrology, Rouen University Hospital, Rouen and INSERM, U1096 Rouen, France; 14 Department of Nephrology, Dialysis and Renal Transplantation, University Hospital, F-63000 Clermont-Ferrand, France; m_tamain@chu-clermontferrand.fr; 15 Department of Nephrology, Dialysis, and Renal Transplantation, University Hospital, F-87000 Limoges, France; marie.essig@inserm.fr; 16 Department of Nephrology, F-14000 Caen University Hospital, Caen, France and the French Registry of Peritoneal Dialysis, Langue Française, F-95300 Pontoise, France; 17 Department of Nephrology and Internal Medicine, Valenciennes General Hospital, F-59300 Valenciennes, France; 18 Department of Nephrology, Dialysis and Renal Transplantation, University Hospital, F-21000 Dijon, France; 19 Department of Nephrology, Orleans Hospital, F-45100 Orleans, France; alexandre.ganea@chr-orleans.fr; 20 Department of Nephrology, Dialysis and Renal Transplantation, Saint-Louis Hospital, AP-HP, Paris and Paris Diderot University, UMR S-1160 alloimmunite-autoimmunite-transplantation (A2T), F-75010 Paris, France; marie-noelle.peraldi@aphp.fr; 21 Nephrology Department, Bichat-Claude Bernard Hospital, APHP, Paris, France. Faculty of Medicine, Paris Diderot University, Sorbonne Paris Cité, F-75018 Paris, France; 22 Department of Nephrology, Duchenne Hospital, F-62200 Boulogne-Sur-Mer, France; 23 Biostatistics Department, Clinical Research and Innovation Department, Amiens University Hospital, F-80054 Amiens, France; 24 Department of Nephrology, Dialysis, and Transplantation, Amiens University Hospital, F-80054 Amiens, France

**Keywords:** anti-GBM disease, ANCA, infection, age, survival

## Abstract

In patients presenting with anti-glomerular basement membrane (GBM) disease with advanced isolated kidney involvement, the benefit of intensive therapy remains controversial due to adverse events, particularly infection. We aim to describe the burden of severe infections (SI) (requiring hospitalization or intravenous antibiotics) and identify predictive factors of SI in a large cohort of patients with anti-GBM disease. Among the 201 patients (median [IQR] age, 53 [30–71] years) included, 74 had pulmonary involvement and 127 isolated glomerulonephritis. A total of 161 SI occurred in 116 patients during the first year after diagnosis. These infections occurred during the early stage of care (median [IQR] time, 13 [8–19] days after diagnosis) with mainly pulmonary (45%), catheter-associated bacteremia (22%) and urinary tract (21%) infections. In multivariable analysis, positive ANCA (HR [95% CI] 1.62 [1.07−2.44]; *p* = 0.02) and age at diagnosis (HR [95% CI] 1.10 [1.00–1.21]; *p* = 0.047) remained independently associated with SI. Age-adjusted severe infection during the first three months was associated with an increased three-year mortality rate (HR [95% CI] 3.13 [1.24–7.88]; *p* = 0.01). Thus, SI is a common early complication in anti-GBM disease, particularly in the elderly and those with positive anti-neutrophil cytoplasmic antibodies (ANCA). No significant association was observed between immunosuppressive strategy and occurrence of SI.

## 1. Introduction

With an estimated annual incidence of 0.5–1 per million in the population, anti-glomerular basement membrane disease (anti-GBM disease) is a rare form of immune complex small vessel vasculitis [1]. This autoimmune disease is mediated by autoantibodies directed against the non-collagenous domain of the α3 chain of type IV collagen [2,3]. The age distribution is bimodal, with one peak in young males and a second peak found predominantly in females in their sixth decade of life [4,5]. The conventional association between rapidly progressive glomerulonephritis and diffuse alveolar hemorrhage (DAH) is predominant in younger patients, whereas older patients show mostly isolated glomerulonephritis and are more likely to be double-positive, presenting with anti-neutrophil cytoplasmic antibodies (ANCA) [6,7,8]. A combination of plasma exchange (PLEX), corticosteroids (CST), and cyclophosphamide (CYC) has improved survival, particularly when the lungs are involved [9,10,11], and some observational studies have suggested the efficacy of rituximab (RTX) as an adjunct to CYC or alone [12,13]. However, kidney outcome is still very poor, with a one-year dialysis-free survival rate ranging from 16 to 37% despite intensive therapy [14,15,16]. For older patients in whom isolated glomerulonephritis is most common, the benefit of intensive treatment remains controversial in the case of anuria or dialysis dependence, particularly regarding a hypothetical increased infectious risk. However, data on severe infections are lacking in patients with anti-GBM disease.

The aim of this study is to describe the burden of severe infections, identify predictive factors, and evaluate the impact on survival in a large French cohort of patients presenting with anti-GBM disease between 1997 and April 2017.

## 2. Materials and Methods

### 2.1. Study Cohort

We performed a retrospective analysis of data from 201 patients diagnosed with anti-GBM disease in the nephrology departments of 22 French centers (19 university and three tertiary hospitals) from January 1997 to December 2017. Patients presenting with clinical manifestations of rapidly progressive glomerulonephritis and with glomerular linear IgG deposits on renal biopsy and/or positive serum anti-GBM antibodies were included. Double-positive patients presenting anti-GBM disease (glomerular linear IgG deposits) and positive ANCA assay results (indirect immunofluorescence and/or an antigen-specific immunoassay) were included in the study. The time of diagnosis was defined as the date of the first detection of anti-GBM by serology or histology. The study protocol was approved by the local independent ethics committee (Amiens, France; reference: TB/LR/2016–91).

### 2.2. Clinical Data

Patients were entered into the study when the diagnosis of anti-GBM disease was established. Clinical and biological data were retrieved from medical records in each center at presentation and during follow-up. C-reactive protein and serum albumin were routinely measured by a liquid-phase immunoassay. The Charlson Comorbidity Index (CCI) was calculated from data on each patient [17]. When performed, kidney biopsies were rated according to the Berden et al. prognostic classification [18]. DAH was defined as the presence of diffuse, bilateral, parenchymal infiltrates on chest imaging, together with either hemoptysis or visual detection of bleeding during bronchoalveolar lavage [19]. Hypoxemic respiratory failure was defined as present when patients required an oxygen flow rate ≥6 L/min to maintain a blood oxygen saturation ≥92%. Standard regimen was defined as the combination of CST+PLEX+IMS (CYC and/or RTX). Alternative regimen was defined as the combination of two treatments among CST, IMS, and PLEX. CST alone or supportive care were also defined as alternative regimens. Severe infections (defined as episodes of infection requiring hospitalization or intravenous antibiotic administration) occurring in the first year after diagnosis were recorded. Dialysis dependency at presentation was defined as the need for renal replacement therapy during the first hospital stay. End-stage renal disease (ESRD) was defined as a Modification of Diet in Renal Disease equation (MDRD) glomerular filtration rate (GFR) ≤15 mL/min/1.73 m^2^ for at least three months. Patient survival was defined as the time from diagnosis to death. Severe infection-free survival was defined as the time from diagnosis to the first severe infection during the first year. The last follow-up corresponded to the patient’s death or the last visit before the end of the study (4 December, 2018). The primary endpoint was the one-year severe infection rate, and the secondary endpoint was the three-year patient mortality rate.

### 2.3. Statistical Analyses

Patient characteristics were summarized as frequency (%) for categorical variables and as mean ± standard deviation (SD) for continuous variables. Risk factors for one-year severe infection and three-year mortality were evaluated using the univariate and multivariable Cox proportional-hazards model, and the results were expressed as hazard ratio (HR) [95% confidence interval (CI)] and *p*-value. Survival was assessed using the Kaplan-Meier method. The median follow-up time was calculated using the reverse Kaplan-Meier method. The threshold for statistical significance was set at *p* < 0.05 in all univariate and multivariable analyses. All statistical analyses were performed using SAS software (version 9.4, SAS Institute Inc., Cary, NC, USA) and R software (version 3.2.3, URL: http://www.R-project.org/).

## 3. Results

### 3.1. Study Population

**Figure 1:**
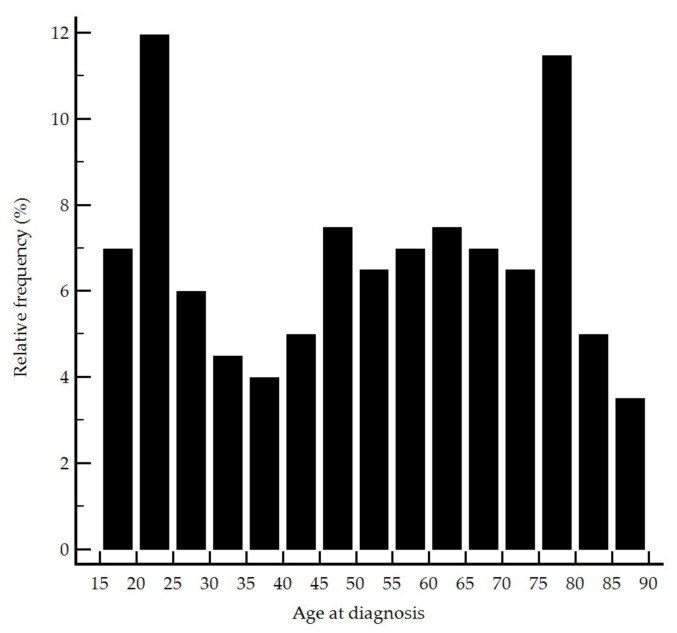
Age distribution of the patients at diagnosis.

A total of 201 patients, including 114 men (57%), were included in the study. The baseline demographic and clinical data by disease presentations are summarized in Table 1. The median [IQR] age at diagnosis was 53 [30–71] years old with peaks at the second and seventh decades (Figure 1).

The median [IQR] peak SCr level was 655 μmol/L [362–1044], and 149 (74%) patients required dialysis at presentation. A total of 74 (37%) patients presented with pulmonary involvement, and 37 (49%) of them experienced hypoxemic respiratory failure of whom 16 (43%) required mechanical ventilation support. Patients with pulmonary involvement were significantly younger compared to patients with isolated glomerulonephritis. The median peak SCr level tended to be lower in this group (573 [276–908] vs. 700 [450–1058] µmol/L; *p* = 0.06). Inflammatory markers were elevated in both groups, but anemia was more marked in patients with pulmonary involvement (p < 0.001). Patients with positive ANCA were significantly older (p < 0.001) and had more severe kidney involvement (median [IQR] peak SCr 859 [549–1242] vs. 585 [288–977] µmol/L; *p* = 0.003) (Supplemental Table S1).

### 3.2. Therapeutic Management

**Figure 2:**
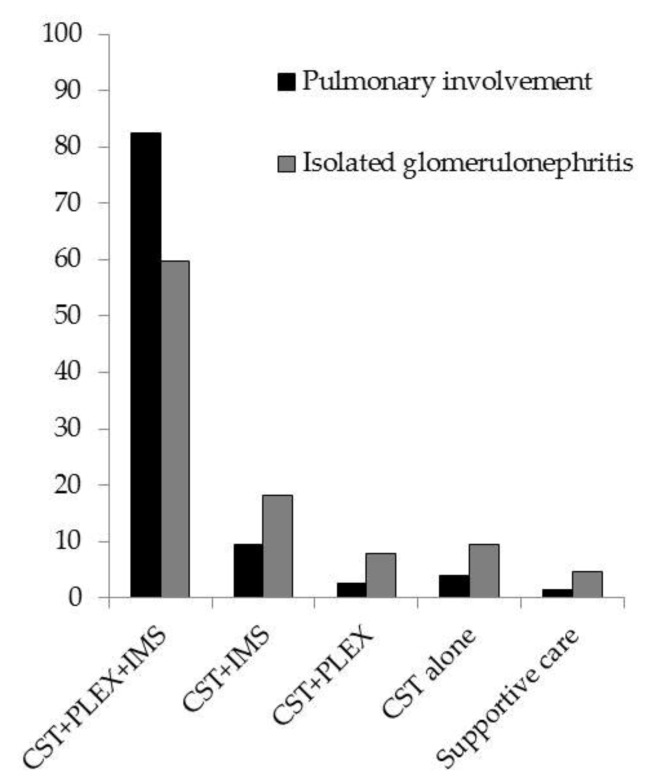
Patient treatment characteristics by disease presentation. Values are expressed as percent. CST: corticosteroids, IMS: immunosuppressive agent (cyclophosphamide and/or rituximab), PLEX: plasma exchange.

Among the 201 patients, 136 (68%) received a standard course of therapy and 65 (32%) received an alternative regimen, including seven (3%) patients receiving only supportive care. Patients with pulmonary involvement were more likely to receive a standard regimen: 61 (82%) patients versus 76 (60%) in patients with isolated glomerulonephritis (*p* < 0.001) (Figure 2).

All 194 patients who were actively treated received a high dose of oral CST preceded by a median of three daily methylprednisolone pulses for 189 of them. The median [IQR] one-month daily dose was 0.8 [0.7–1.0] mg/kg. A total of 149 (77%) patients received a median [IQR] of 12 [7–15] PLEX sessions. The proportion of patients receiving PLEX and the number of sessions was significantly higher in patients with pulmonary involvement. Immunosuppression was initiated in 166 patients, mostly with CYC (157 cases, 80% intravenously), with a median [IQR] one-month cumulative dose of 28 [16–40] mg/kg. Thirteen patients received RTX therapy, combined with CYC in five cases. Patients with pulmonary involvement received immunosuppressive treatment more promptly compared to those with isolated glomerulonephritis (68 (92%) vs. 98 (77%); *p* = 0.008). The median [IQR] six-month cumulative dose of CYC was also higher in this group (86 [41–120] vs. 56 [31–90] mg/kg; *p* = 0.02). Co-trimoxazole prophylaxis (CTZ) was used in 138 (71%) of the actively treated patients.

Among 191 patients alive at three months, 160 (84%) were under immunosuppressive therapy, mostly as a combination of CST+CYC (57%). At six months, 115 patients were receiving CST at a median daily dose of 10 [5–15] mg. At one year, 43 patients were receiving a maintenance treatment, CST alone in 23 cases. The renal response to the treatment was poor in patients who required dialysis at presentation, with only 10 (6.7%) patients weaned off dialysis at one year. The renal prognosis was better in the 52 (26%) patients free of dialysis at diagnosis, with 10 (19%) patients developing end-stage renal disease (nine on dialysis and one transplanted) and 42 (81%) patients conserving relatively good renal function. The median [IQR] SCr level was 140 [86–216] µmol/L and the GFR was 50 [26–89] mL/min at 12 months.

### 3.3. Severe Infections

During the first year of follow-up, 161 severe infections were recorded in 116 (57.7%) patients, resulting in an incidence rate of 58 cases/100 patient-years. Patients having at least one severe infection were significantly older (57 [40–73] vs. 45 [22–64] years old; *p* = 0.001) and were more frequently ANCA positive (34 vs. 16%; *p* = 0.004). Dose and duration of immunosuppressive therapies was similar between those with and without severe infection. Baseline patient demographic and clinical data by infection are summarized in Table 2.

These infections mostly occurred during the early stage of care, with a median [IQR] time to the first infection of 13 [8–19] days after diagnosis. The main sources of infection were lung (45%), catheter (22%), and urinary tract (21%). Causative organisms were identified in 86 infections (53%): *Staphylococcus aureus* (*n* = 23) and *Escherichia coli* (*n* = 19) were the most common, followed by *Streptococcus pneumoniae* (*n* = 8), *Klebsiella pneumoniae* (*n* = 6), and *Pseudomonas aeruginosa* (*n* = 5). Resistant strains were rarely identified, with four extended-spectrum beta-lactamase positive (ESBL) *E. coli*, 2 methicillin-resistant *S. aureus,* and one ESBL *K. pneumonia.* Fungal infections were rare and consisted of six (4%) *Pneumocystis* infections. Patients with lung involvement were more susceptible to pulmonary infections accounting for 55% of the SI observed in this group, compared to 39% in patients with isolated glomerulonephritis; *p* = 0.04. Patients with severe infections had more hypoxemic respiratory failure (25% vs. 9%; *p* = 0.005), and 29 (25%) experienced septic shock.

In univariate analysis, age at diagnosis, Charlson Comorbidity Index, Performance Status, and positive ANCA at diagnosis were associated with one-year severe infection occurrence. In multivariable analysis, only age at diagnosis (HR 1.10; 95% confidence interval ([95% CI]) [1.00–1.21]; *p* = 0.047) and positive ANCA (HR 1.62; [95% CI] [1.07–2.44]; *p* = 0.02) remained independently associated with the occurrence of severe infection within the first year (Table 3).

**Figure 3:**
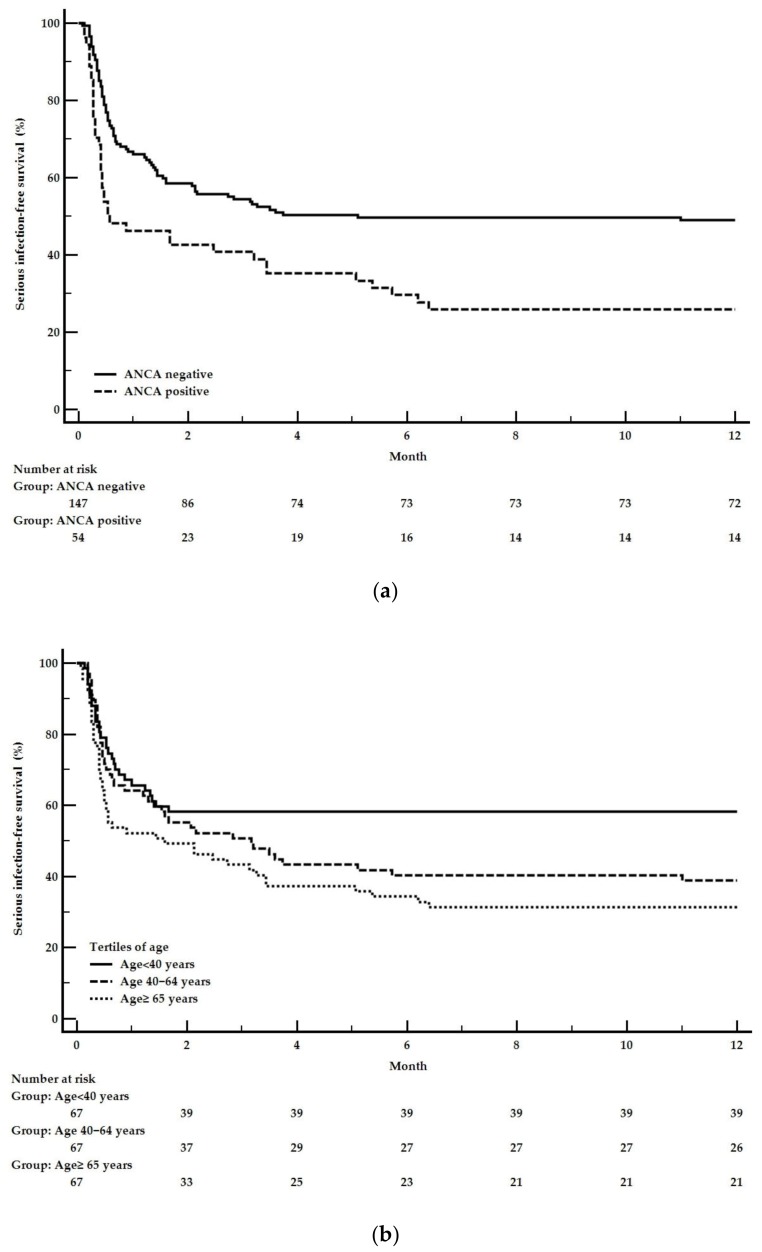
Kaplan-Meier curves for serious infection-free one-year survival as a function of independent predictors: (**a**) Positive ANCA. Logrank *p*-value = 0.003. (**b**) Age at diagnosis by tertile. Logrank *p*-value = 0.015. Compared to the lowest age tertile (<40), the second age tertile (40–64) had a hazard ratio [95% CI] of 1.54 [0.95–2.49], and the third age tertile (≥65) had a hazard ratio [95% CI] of 1.98 [1.23–3.16].

One-year severe infection-free survival, as a function of the two independent predictors, is presented in Figure 3.

### 3.4. Patient Survival

The median [95% CI] follow-up survival rate was 73 [63–93] months. The one-, three-, and five-year [95% CI] patient-survival rates were respectively 92% [87–96], 89% [85–93], and 79% [73–85]. Overall, 25 patients died within the first three years. Most of these deaths occurred during the first year (19 cases), almost exclusively in older patients. Infection and cardiovascular events were the main causes of death (Supplemental Table S2). When combined with age at diagnosis in a multivariable Cox analysis, CCI (HR [95% CI] 1.39 [1.08–1.80]; *p* = 0.01), ≥2 cardiovascular disease risk factors (HR [95% CI] 3.49 [1.51–8.05]; *p* = 0.003), and the occurrence of a severe infection during the first three months (HR [95% CI] = 3.13 [1.24–7.88]; *p* = 0.01) remained independently associated with three-year mortality (Table 4).

**Figure 4:**
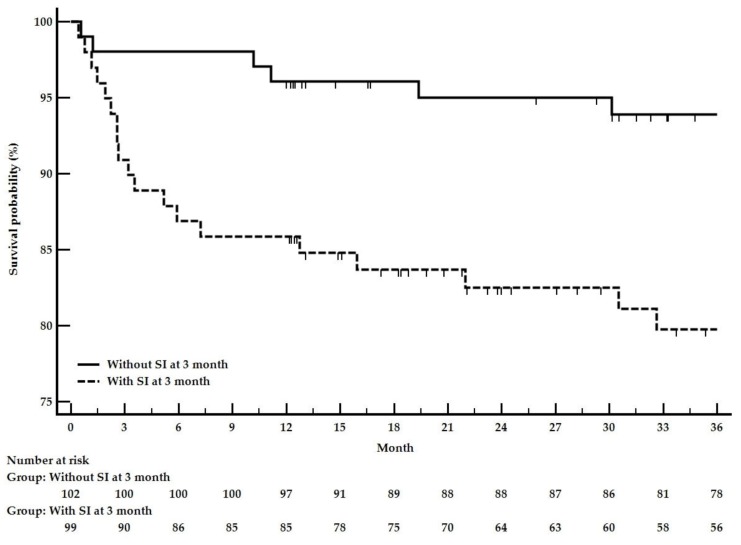
Kaplan-Meier curves for three-year survival as a function of occurrence of a severe infection in the first three months of follow-up. Logrank *p*-value = 0.003.

Three-year survival as a function of the occurrence of severe infection at three months is presented in Figure 4.

## 4. Discussion

The beneficial effect of active treatment on patient survival was demonstrated forty years ago [9,20]. Anti-GBM disease was considered as rapidly fatal before implementation of immunosuppressive therapies [21,22]. Despite poor kidney presentation, most patients in the present study were actively treated. In these conditions, the one-year survival rate was about 92% [87–96], similar to results of recent studies [14,16]. However, severe infections were frequent during the early phase of the disease and were associated with substantial morbidity and a reduced three-year survival rate. The lungs, the urinary tract, and catheters were the main sites of infection, most often caused by *Staphylococcus aureus* and *Escherichia coli* when pathogens were identified. Advanced age at diagnosis and positive ANCA were independently associated with an increased severe infection rate during the first stage of care.

In a cohort of 122 patients, Huart et al. found that infection accounts for almost 50% of deaths occurring in the first year of follow-up, similar to our population [16]. In a recent study, Gu et al. described the high prevalence of infection, with 74 of 140 Chinese patients with anti-GBM disease experiencing at least one infectious episode [23]. Of these, 58 patients developed infections at the onset of the disease, mainly located in the respiratory tract. The predominance of pulmonary infection was also observed in our cohort and was more frequent in patients presenting with DAH. Alveolar injury is most likely to decrease the resistance of the mucous membranes to pathogens, contributing to the development of superinfection, as observed in other systemic diseases [24,25,26]. Similar to the cohort described by Gu et al., the CRP level at diagnosis in our study was significantly higher in patients with infections, suggesting, as reported in previous studies, that latent infection may play a role in initiating disease [27,28,29].

No statistical association was observed between immunosuppressive strategy and occurrence of SI, partly due to the precocity of the SI and because immunosuppressive treatment is frequently reduced following an infection [30]. Whether an immunosuppressant-sparing strategy could help to reduce the burden of SI in this population remains to be determined. The association between CST exposure and severe infection has previously been established [31,32,33]. Nevertheless, advanced age at diagnosis was a major contributor to SI in our study. The increased infectious risk and poor kidney outcome we observed raises the question of whether dialysis-dependent older patients with isolated kidney involvement, would benefit from aggressive immunosuppressive treatment. Immunosuppressant-sparing strategies should be strongly considered in these patients, and a wait-and-see approach could be discussed for the most vulnerable patients. However, the potential reduction of adverse events needs to be balanced with the potential risk of delayed lung involvement, notably in patients presenting with a high level of circulating anti-GBM antibodies.

More than 50% and almost 25% of our patients were under CST at 6 and 12 months, respectively. The benefit of extended (beyond six months) immunosuppressive treatment has not been established in anti-GBM disease and is not currently recommended [34]. Co-trimoxazole prophylaxis was not associated with a reduced rate of SI in our cohort. However, its use has been shown to be protective in other similar conditions such as ANCA-associated vasculitis (AAV) [35,36].

Patients with positive ANCA were more likely to develop SI, even when the analysis was restricted to ANCA with specificity for proteinase 3 and/or myeloperoxidase. To our knowledge, being positive for ANCA has not yet been linked to infection in anti-GBM disease. However, it is well known that infection is the main cause of early death in AAV [30,35]. Some researchers have observed that, in addition to their inflammatory profile, AAV patients have neutrophils with a high apoptosis rate and reduced activation capacity [37], which may contribute to their diminished ability to contain or eliminate bacterial pathogens. Some researchers recently detected inhibitory anti-peroxidasin autoantibodies present in serum from patients before and at the onset of anti-GBM disease [38]. Peroxydasin shares structural homology with MPO, and anti-peroxidasin autoantibodies cross-react in vitro with MPO, which could account for some “false” MPO positive patients [39]. Peroxydasin is involved in host defense by directly binding with and killing gram negative bacteria [40]. One might hypothesize that the inhibitory activity of anti-peroxidasin autoantibodies may contribute to the higher rate of bacterial infection observed in double-positive patients. However, ANCA may be positive in infectious processes, particularly in bacterial endocarditis [41,42,43,44]. The link between ANCA and infection is complex and would require further investigations, but previous publications have hypothesized that ANCA may be induced by antigenic stimulation secondary to neutrophilic enzymes released at the site of infection [43].

The main limitation of our study is its retrospective design, which could lead to information bias. However, the data analyzed were recorded during the first stage of care. The study was restricted and performed in nephrology departments, limiting the inclusion of patients with isolated pulmonary involvement. A large number of the SI cases were diagnosed during the first hospital stay, thus were not the primary cause of hospitalization. The lack of identification of the causative pathogens in almost half of the cases of SI may suggest that the incidence could have been overestimated. However, pulmonary infections were predominant, and the diagnostic invasive specimen yield generally does not exceed 60% in immunocompromised patients [45]. Most of the previous studies have focused on the kidneys and overall survival; therefore, few comparable data are available for anti-GBM disease.

## 5. Conclusion

In conclusion, despite a very poor kidney prognosis, patients with anti-GBM disease have a good survival rate. About 57.7% of the patients had SI during the first 12 months of care, particularly in older patients and in patients who were ANCA positive at diagnosis. These data suggest that comprehensive investigations and careful monitoring for bacterial infection should be routinely performed at the early stage of patient management along with the initiation of immunosuppression to reduce infection-related morbidity.

## Figures and Tables

**Table 1:** Patient characteristics at diagnosis.

	Total (*n* = 201)	Pulmonary Involvement (*n* = 74)	Isolated Glomerulonephritis (*n* = 127)	*p*-Value
Age	53 [30–71]	43 [23–63]	59 [39–75]	0.001
Male	114 (57)	44 (60)	70 (55)	0.55
**Comorbidities**				
Diabetes mellitus	14 (7)	3 (4)	11 (9)	0.22
Chronic arterial hypertension	71 (35)	22 (30)	49 (39)	0.21
Dyslipidemia	41(20)	7 (10)	34 (27)	0.003
Tobacco use	85 (42)	45 (61)	40 (32)	<0.001
Cardiovascular diseases	15 (7)	5 (7)	10 (8)	0.66
Cancer	22 (11)	7 (10)	15 (12)	0.61
Charlson Comorbidity Index	0 [0–1]	0 [0–0]	0 [0–1]	0.006
Performance Status	1 [1–2]	1 [1–2]	1 [1–2]	0.07
**Antibodies**				
Anti-GBM	178 (89)	69 (93)	109 (86)	0.11
ANCA	54 (26)	18 (23)	36 (28)	0.53
-MPO	37 (68)	12 (67)	25 (69)	0.95
-PR3	12 (22)	4 (22)	8 (22)	0.92
**Kidney Involvement**				
Oligoanuria	128 (64)	46 (62)	82 (65)	0.73
Peak SCr (µmol/L)	655 [362–1044]	573 [276–908]	700 [450–1058]	0.06
Peak SCr ≥ 500 µmol/L	134 (67)	44 (60)	90 (71)	0.10
Proteinuria ≥ 1.5 (g/j)	148 (73)	59 (80)	89 (70)	0.14
Needed dialysis at initial presentation	149 (74)	53 (72)	96 (76)	0.54
Kidney biopsy findings	179 (89)	59 (80)	120 (95)	0.001
-Focal	16 (9)	8 (13)	8 (7)	0.13
-Cellular	116 (64)	34 (58)	82 (68)	0.18
-Fibrous	34 (19)	10 (17)	24 (20)	0.62
-Mixed	13 (7)	7 (12)	6 (5)	0.09
**Inflammatory Markers**				
Serum albumin (g/L)	26.2 [23.0–30.0]	25.6 [22.0–30.0]	26.7 [23.3–32.0]	0.19
Leukocytes (cells/mm3)	11.0 [8.1–13.3]	11.7 [8.3–14.1]	10.5 [7.9–12.9]	0.18
Hemoglobin (g/dL)	8.6 [7.5–9.9]	7.7 [7.0–9.0]	9.0 [8.0–10.4]	<0.001
C-reactive protein (mg/L)	102 [33–170]	101 [40–160]	103 [32–182]	0.87

Values are expressed as median [interquartile range] or number (percent). Anti-GBM: anti-glomerular basement membrane antibodies, ANCA: anti-neutrophil cytoplasmic antibodies, MPO: myeloperoxidase, PR3: proteinase 3, SCr: serum creatinine.

**Table 2:** Patient characteristics at diagnosis by infectious status.

	Total (*n* = 201)	Severe Infection (*n* = 116)	No Severe Infections (*n* = 85)	*p*-Value
Age	53 [30–71]	57.5 [40–73]	45 [22–64]	0.001
Male	114 (57)	71 (61)	43 (51)	0.13
**Comorbidities**				
Diabetes mellitus	14 (7)	12 (10)	2 (2)	0.03
Chronic arterial hypertension	71 (35)	50 (43)	21 (25)	0.007
Charlson Comorbidity Index	1 [0–4]	2 [1–4]	1 [0–2]	0.001
Performance Status	1 [1–2]	2 [1–2]	1 [1–2]	0.08
**Antibodies**				
Anti-GBM	178 (89)	102 (88)	76 (89)	0.74
ANCA	54 (27)	40 (34)	14 (16)	0.004
-MPO	38 (70)	28 (70)	10 (71)	0.92
-PR3	13 (24)	10 (25)	3 (21)	0.79
**Kidney Involvement**				
Oligoanuria	128 (64)	78 (67)	50 (59)	0.22
Peak SCr (µmol/L)	655 [362–1044]	663 [400–1077]	651 [313–1000]	0.63
Peak SCr ≥500 µmol/L	134 (66)	80 (69)	54 (64)	0.42
Needed dialysis at initial presentation	149 (74)	89 (77)	60 (71)	0.33
**Lung Involvement**				
Alveolar hemorrhage	74 (37)	42 (36)	32 (38)	0.83
Hypoxemic respiratory failure	37 (18)	29 (25)	8 (9)	0.005
**Inflammatory Markers**				
Serum albumin (g/L)	26.2 [23.0–30.0]	26.1 [22.9–30.0]	26.7 [22.9–30.8]	0.64
Leukocytes (cells/mm3)	11.0 [8.1–13.3]	11.0 [7.9–13.4]	10.7 [8.4–12.5]	0.89
Hemoglobin (g/dL)	8.6 [7.5–9.9]	8.5 [7.4–10.1]	8.6 [7.6–9.7]	0.86
C-reactive protein (mg/L)	102 [33–170]	110 [45–183]	84 [25–140]	0.047
Therapeutic management *				
Methylprednisolone pulses	189 (97)	107 (97)	82 (98)	0.88
CST Daily 6-month dose (mg) #	10 [5–15]	10 [10–15]	10 [9–20]	0.58
Plasma exchange	149 (77)	87 (79)	62 (74)	0.39
Number of sessions	12 [7–15]	12 [7–14]	12 [8–15]	0.73
Cyclophosphamide	157 (81)	91 (83)	66 (79)	0.47
Cumulative 6-month dose (mg/kg)	63 [35–101]	62 [38–97]	67 [28–103]	0.29
Co-trimoxazole prophylaxis	138 (71)	79 (72)	59 (70)	0.84

Values are expressed as median [interquartile range] or number (percent). Anti-GBM: anti-glomerular basement membrane antibodies, ANCA: anti-neutrophil cytoplasmic antibodies, MPO: myeloperoxidase, PR3: proteinase 3, SCr: serum creatinine; CST: corticosteroids. * Population limited to the 194 patients actively treated. #115 patients under CST at six months. Severe infections (*n* = 67), No severe infections (*n* = 48).

**Table 3:** Factors associated with severe infection during the first year of follow-up.

	Univariate Analysis		Multivariable Analysis	
Variable	HR [95% CI]	*p*-Value	HR [95% CI]	*p*-Value
Age *	1.14 [1.04–1.24]	0.004	1.10 [1.00–1.21]	0.047
Diabetes mellitus	1.68 [0.92–3.10]	0.09		
Charlson Comorbidity Index	1.17 [1.01–1.35]	0.032		
Performance Status	1.41 [1.08–1.85]	0.013		
SCr > 500 µmol/L	1.24 [0.84–1.85]	0.28		
Dialysis	1.31 [0.85–2.03]	0.23		
Alveolar hemorrhage	1.00 [0.69–1.46]	0.99		
ANCA	1.88 [1.21–2.92]	0.001	1.62 [1.07–2.44]	0.02
Serum albumin	0.99 [0.96–1.02]	0.63		
Hemoglobin	1.06 [0.97–1.15]	0.19		
Standard therapy	0.94 [0.64–1.39]	0.77		
Number of PLEX sessions	1.00 [0.98–1.02]	0.99		
Methylprednisolone pulses	0.83 [0.39–1.78]	0.63		
Cumulative CYC dose at M1	0.85 [0.55–1.33]	0.48		
Co-trimoxazole prophylaxis	0.88 [0.58–1.32]	0.53		

Each variable with a univariate *p* < 0.05 was added into the multivariable Cox regression analysis. HR: hazard ratio, CI: confidence interval, SCr: serum creatinine, CYC: cyclophosphamide, PLEX: plasma exchange. Variables included in the backward multivariable model: age, Charlson Comorbidity Index, Performance Status, positive ANCA. * for each increase of 10 years.

**Table 4:** Factors associated with 3-year mortality.

	Univariate Analysis		Age-Adjusted *	
Variable	HR [95% CI]	*p*-Value	HR [95% CI]	*p*-Value
Age	1.05 [1.03–1.08]	<0.001	–	≤0.004
Female	1.23 [0.56–2.69]	0.60		
≥2 CVD risk factors	5.78 [2.55–13.11]	<0.001	3.49 [1.51–8.05]	0.003
Charlson Comorbidity Index	1.73 [1.41–2.13]	<0.001	1.39 [1.08–1.80]	0.01
Performance Status	2.11 [1.21–3.73]	0.009		
SCr >500 µmol/L	2.02 [0.75–5.40]	0.16		
Dialysis	4.23 [0.99–17.93]	0.05		
Alveolar hemorrhage	0.89 [0.94–2.14]	0.89		
ANCA positivity	1.01 [0.42–2.43]	0.97		
Serum albumin	0.95 [0.89–1.02]	0.20		
Hemoglobin	1.06 [0.98–1.14]	0.10		
Standard therapy	0.83 [0.36–1.89]	0.96		
Co-trimoxazole prophylaxis	0.78 [0.35–1.78]	0.56		
Severe infection at 3 months	3.60 [1.45–9.08]	0.006	3.13 [1.24–7.88]	0.01

* Given the limited number of events (25 deaths), each variable with a univariate *p* < 0.05 was added as the second variable in a multivariable Cox regression analysis that included age at presentation. Age remained significant in each analysis (*p* ≤ 0.004). HR: hazard ratio, CI: confidence interval, CVD: cardiovascular disease, SCr: serum creatinine.

## Supplementary Materials

The following are available online at https://www.mdpi.com/2077-0383/9/3/698/s1, Table S1: Patient characteristics based on ANCA status, Table S2: Causes of death within the first three years of follow-up.
