# Supplementary material for: Severe Infection in Anti-Glomerular Basement Membrane Disease: A Retrospective Multicenter French Study
Source: J Clin Med. 2020 Mar 4;9(3):698. doi: 10.3390/jcm9030698 (PMC7141378; doi:10.3390/jcm9030698)
Supplement: Supplementary file 1 [file jcm-09-00698-s001.zip › Supplemental material/supplemental Table 1.pdf]

Supplemental Table 1. Patient characteristics based on ANCA status

	**Total** (n=201)	**ANCA positive** (n=54)	**ANCA negative** (n=147)	***p*-value**
Age	53 [30–71]	70 [56-76]	46 [26–64]	<0.0001
Male	114 (57)	30 (56)	84 (57)	0.84
**Comorbidities**				
**Charlson Comorbidity Index**	0 [0–1]	1 [0-1]	0 [0-1]	<0.0001
**Performance Status**	1 [1–2]	2 [1-2]	1 [1-2]	0.002
Tobacco use	85 (42)	13 (24)	72 (49)	0.002
**Kidney involvement**				
Oligoanuria	128 (64)	40 (74)	88 (60)	0.06
Peak SCr (µmol/L)	655 [362–1044]	859 [549-1242]	585 [288-977]	0.003
Needed dialysis at initial presentation	149 (74)	46 (85)	103 (70)	0.03
**Kidney biopsy findings**	180 (90)	46 (85)	134 (91)	0.22
-Focal	16 (9)	0 (0)	16 (12)	0.01
-Cellular	116 (65)	28 (61)	88 (66)	0.56
-Fibrous	35 (19)	14 (30)	21 (15)	0.03
-Mixed	13 (7)	4 (9)	9 (7)	0.66
**Lung involvement**				
Alveolar hemorrhage	74 (37)	18 (33)	56 (38)	0.54
Hypoxemic respiratory failure	37 (18)	6 (11)	31 (21)	0.11
**Inflammatory markers**				
Serum albumin (g/L)	26.2 [23.0–30.0]	26 [22.4–30.0]	26.6 [23.0–30.0]	0.53
Leukocytes (cells/mm^3)	11.0 [8.1–13.3]	11.5 [8.0–13.4]	10.7 [8.1–12.8]	0.61
Hemoglobin (g/dL)	8.6 [7.5–9.9]	9 [7.7–10.3]	8.4 [7.4–9.7]	0.06
C-reactive protein (mg/L)	102 [33–170]	110 [54–169]	98 [25–172]	0.19
**Therapeutic management**				
Standard therapy	137 (68)	31 (57)	106 (72)	0.048
Therapy M6	115 (62*)	28 (57*)	87 (64*)	0.53
Therapy M12	43 (24#)	11 (23#)	32 (25#)	0.83
**Co-trimoxazole prophylaxis**	138 (69)	39 (72)	99 (67)	0.51
**Severe infections** §	116 (58)	40 (74)	76 (52)	0.0045
**Septic shock**	29 (25$)	10 (25$)	19 (25$)	1

Values are expressed as median [interquartile range] or number (percent). Anti-GBM, anti-glomerular basement membrane antibodies; ANCA, anti-neutrophil cytoplasmic antibodies; MPO, myeloperoxidase; PR3, proteinase 3; SCr, serum creatinine; Standard therapy defined as the association of corticosteroid (CST)+ plasma exchange (PLEX)+cyclophosphamide (CYC) and/or Rituximab (RTX). * reported as a percentage of patient alive (n=185 with 49 ANCA positive and 136 ANCA negative). # reported as a percentage of patient alive (n=177 with 48 ANCA positive and 129 ANCA negative). § number of patients presented at least one serious infection during the first year. $ reported as a percentage of the number of patients.
