# Supplementary material for: Severe Infection in Anti-Glomerular Basement Membrane Disease: A Retrospective Multicenter French Study
Source: J Clin Med. 2020 Mar 4;9(3):698. doi: 10.3390/jcm9030698 (PMC7141378; doi:10.3390/jcm9030698)
Supplement: Supplementary file 1 [file jcm-09-00698-s001.zip › Supplemental material/Supplemental Table 2.pdf]

Supplemental Table 2: Causes of death within the first 3 years of follow-up

Age	Time interval (months)	Cause	Specific cause
77	0.43	**Infection**	*S. aureus* septic shock
72	0.57	**Cardiovascular**	Subarachnoid hemorrhage
56	0.77	**Infection + vasculitis**	Infected diffuse alveolar hemorrhage (ARDS)
89	1.13	**Infection**	Methicillin-resistant *S. aureus* septic shock
88	1.20	**Treatment limitation**	Discontinuation of dialysis
86	1.47	**Malignancy**	Lung and prostate cancer
79	1.90	**Infection**	*S. aureus* septic shock
66	2.23	**Cardiovascular**	Cerebellar hemorrhagic stroke
38	2.57	**Cardiovascular**	Undetermined cardiac arrest.
76	2.57	**Infection + vasculitis**	Infected diffuse alveolar hemorrhage (ARDS)
78,5	2.63	**Infection**	Hemorrhagic and septic shock
80	3.20	**Cardiovascular**	Ventricular rhythm disorder
67	3.53	**Infection**	Herpes simplex encephalitis
82	5.17	**Treatment limitation**	Discontinuation of dialysis
83	5.90	**Infection**	Pulmonary septic shock.
47	7.23	**Cardiovascular**	Extensive ischemic stroke
77	10.17	**Treatment limitation**	Discontinuation of dialysis
20	11.13	**Metabolic**	Hyperkalemia
82	12.73	**Malignancy**	Metastatic colon cancer
52	15.93	**Infection**	Septic shock
42	19.37	**Cardiovascular**	Congestive heart failure
88	21.97	**Infection**	Gut-derived septic shock
59	30.13	**Malignancy**	Colon cancer
67	30.50	**Cardiovascular**	Acute coronary syndrome
67	32.63	**Cardiovascular**	Ventricular rhythm disorder

Values are expressed as number. *S. aureus* : *Staphylococcus aureus*, ARDS: acute respiratory distress syndrome
